# Phytochemical screening of *Prunus avium* for its antioxidative and anti-mutagenic potential against DMBA-induced hepatocarcinogenesis

**DOI:** 10.3389/fnut.2023.1132356

**Published:** 2023-05-12

**Authors:** Raakia Anam Saeed, Muhammad Issa Khan, Masood Sadiq Butt, Muhammad Naeem Faisal

**Affiliations:** ^1^National Institute of Food Science and Technology, University of Agricultural Faisalabad, Faisalabad, Pakistan; ^2^Institute of Pharmacy, Physiology, and Pharmacology, University of Agriculture Faisalabad, Faisalabad, Pakistan

**Keywords:** hepatocarcinoma, Prunus avium, chemo-preventive, mRNA expression, ATM signaling

## Abstract

**Scope:**

*Prunus avium* fruit is the richer source of phenolics known to exert anticancer and anti-invasive activities. The study aimed at elucidating antiproliferative and chemo-preventive potential of sweet cherries (*P. avium*) against the *in vivo* hepatocarcinoma model.

**Methods and results:**

The quantification of ultrasound-assisted extract (UAE) of *P. avium* depicted anthocyanins, ferulic acid, gallic acid, quercetin, syringic acid and p- and m-coumaric acids as major phytochemicals. The hepatocarcinoma (HCC) was induced in rats through intraperitoneal administration of DMBA (20 mg/kg B.W) once a week for the period of eight weeks. The intragastric administration of *P. avium* UAE, as cotreatment (500 mg/Kg B.W) to treatment group, significantly (*p* < 0.01) attenuated the raised serum alpha-fetoprotein (AFP), carcinoembryonic antigen (CEA), alanine aminotransferase (ALT), aspartate aminotransferase (AST), lactate dehydrogenase (LDH) as well as total oxidative stress (TOS) and enhanced total antioxidant capacity TAOC in contrast to diseased rats. Moreover, microscopic examination of hepatic tissues confirmed the pleomorphism, nests of neoplastic hepatocytes and necrosis in HCC-bearing rats as compared to extract-fed rats, where these necrotic changes were suppressed. Besides, qRT-PCR analysis of hepatic tissues demonstrated the higher mRNA expression of CHEK1, CHEK2 and P21/CDKN1α genes, while downexpression of ATM gene in extract fed rats, further denoting the anti-mutagenic potential.

**Conclusion:**

Consequently, the polyphenol-rich sweet cherries UAE exhibited antiproliferative and chemo-preventive potential by reducing tumor biomarkers, serum transaminases and oxidative stress, as well as enhancing antioxidant status. It further upregulated the downstream targets of ATM signaling cascade.

## Graphical Abstract

Practical application: The present work quantified the phytochemicals in *P. avium* fruit extract and explored its anticancer and chemotherapeutic potential using an *in vivo* hepatocarcinoma model. Conclusively, *P. avium* effectively attenuated the tumor biomarkers and upregulated the expression of genes involved in the DNA repair process. Therefore, *P. avium* inclusion in daily diet may improve general health and prevent degenerative diseases. Moreover, therapeutic potential of sweet cherries and its isolated bioactive metabolites against hepatic carcinoma can explore after clinical trials and standardization in higher animals. 
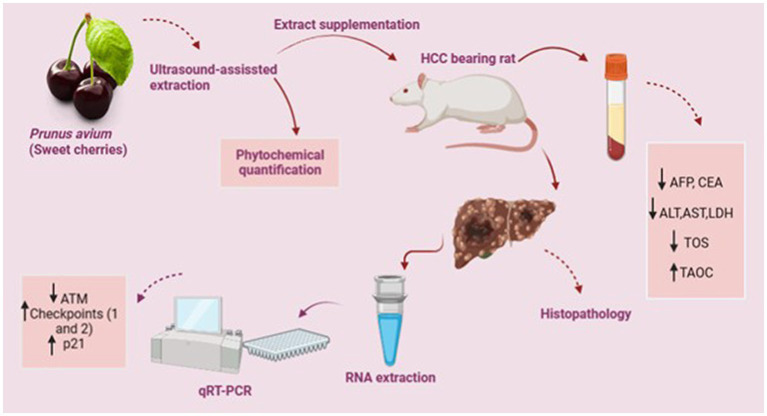

## 1. Introduction

Cancer is characterized by rapid growth, proliferation, and invasion of malignant cells to other body tissues and organs. It has numerous and complex eventual causes; however, these are partially understood. However, aberrant gene expression and altered gene functionality are significant features of carcinogenesis (1). Hepatocarcinoma (HCC), one of the most lethal cancers (below 9% survival rate), resulted in the mortality of approximately 830,000 individuals in 2020. Viruses (hepatitis B or C), nitrosamines, or other carcinogens, aflatoxins, cirrhosis, non-alcoholic and alcoholic liver disease are responsible for its onset (2). Early detection results in curative surgical treatments, but diagnosis is difficult in most cases and only occurs at an advanced stage leading to death (3).

The demand for novel, safer, more efficient, and specific chemotherapeutic agents for cancer prevention is rising. Various epidemiologic studies suggested protective role of vegetables and fruit consumption against cancer due to bioactive compounds (4–9). Among natural molecules, phenolic acids, flavan-3-o, and anthocyanins have been widely studied for their anti-inflammatory, antioxidative, and anti-mutagenic potentials. They further inhibit cancer through modulation of cellular metabolism, induction of cell cycle arrest, inhibition of cellular growth and differentiation, and control of genes linked with tumor cell growth (10–12). This anti-mutagenic potential of phenolic compounds is associated with its enhanced antioxidant status. As evident, tumor cells generate more reactive oxygen species (ROS) and use their signals for initiating proliferation and other events involved in carcinogenesis progression. Phenolic substances mitigate the ROS levels in turn and influence the events involved in the progression of tumor cells (13, 14). Thus, natural products, richer sources of these bioactive molecules, such as sweet cherries, are the target of more profound and extensive studies aiming at discovering their biological capabilities. *Prunus avium* Linnaeus (sweet cherries), colorful perishable fruits belonging to the Rosaceae family, are widely appreciated due to taste, aroma, and nutritional value (15). Besides supplying essential micro and macronutrients, cherries possess significantly higher phytochemical content, such as anthocyanins, hydroxycinnamates and flavin-3-ols, etc. (16).

Owing to the presence of bioactive substances in a relatively higher proportion, it is not unanticipated that the consumption of cherries promotes general health and prevents or cures several degenerative ailments (17–19). Also, several studies suggested the anti-inflammatory potential of sweet cherries through the inhibition of cyclooxygenases (COX-1 and COX-2), prostaglandin E2 (PDE2), interleukin 6 (IL-6) and TNF-α (tumor necrosis factor-alpha), endothelin-1 and plasminogen activator inhibitor-1. This inhibitory effect is further related to the downregulation of mitogen-activated protein kinase (MAPK) and upstreaming of IL-2 and 4 (20, 21). Moreover, some peculiar anticancer effects include arresting the cell cycle, apoptosis induction, inhibiting oxidative stress-induced DNA damage, angiogenesis, and activating detoxification enzymes (phase II enzymes) (22). The intake of sweet cherries reduced the cellular growth *via* suppression of Akt (protein kinase B) and PLCγ-1 (Phospholipase C gamma 1) activation followed by enhanced Bax/Bcl-2 ratio consequently leading to activation of intrinsic and extrinsic apoptotic processes (23). Furthermore, sweet cherries anthocyanins exerted chemotherapeutic impact by downstreaming the mRNA expression of several metastatic biomarkers (Sp1, Sp4 and vascular cell adhesion molecule 1) (24).

Many plant species have already been employed for preventing or delaying the onset of cancer due to less or no toxicity (25–29). Moreover, apart from imposing financial burdens, chemotherapeutic agents are barely free of detrimental effects. Thus, alternative options have upsurged the researchers’ interest in utilizing indigenous therapies for cancer prevention, i.e., food antioxidants and plant-based drugs. Regrettably, only a few studies have explicated the anticancer and antiproliferative potential of *P. avium* against human colon cancer, prostate cancer (30), breast cancer (23, 24), as well as stomach (MKN45) cells (23, 24, 31–34). Also, a recent study established the hepatoprotective role of sweet cherries in HepG2 cells (35). To the best of our knowledge, no systematic scientific studies have been conducted regarding the *in vivo* impact of sweet cherries on HCC progression and proliferation though one animal study assessed the impact of sweet cherries supplementation on hepatic steatosis (36). Thus, apart from exploring the phytochemical content and antioxidant potential of *P. avium*, the current *in vivo* study was designed to assess the anti-tumorigenic potential of *P. avium* against chemically induced hepatocarcinoma in the animal model.

## 2. Materials and methods

*Prunus avium* (sweet cherries) fruits were purchased from a local supplier in Pakistan, and authentication of the it was carried out at the Institute of Horticultural Sciences, University of Agriculture, Faisalabad. All the required analytical grade chemicals and kits were purchased from Sigma-Aldrich (St. Louis, MO, United States), Merck, and Fisher Scientific (CHEMTREC®, United States).

### 2.1. Preparation of ultrasound-assisted extract of *Prunus avium*

The vacuum-dried *P. avium* samples (5 g) were extracted with 43% acidified ethanol (0.1% HCl) with a solid-to-liquid ratio of 1 g/15 mL using a sonicator as described by Milić, Daničić (37) with slight modifications. The volumetric flasks containing solutions were placed in an ultrasonic bath (Elmasonic E30H, GmbH, Germany) at a frequency of 30 kHz for 42 min at 54°C. Afterwards, the mixtures were centrifuged at 9000 rpm, 4°C for 10 min (MPW-352R, refrigerated laboratory centrifuge, United States). After the filtration of the extract with a vacuum filter, subsequent ethanol evaporation was carried out using a rotary evaporator under controlled conditions, i.e., 40°C temperature and 0.1 MPa pressure (EYELA Rotary Vacuum Evaporator N-N Series). The obtained extract was then stored at -20°C till further usage.

### 2.2. Phytochemical screening

#### 2.2.1. *In vitro* antioxidant assay

##### 2.2.1.1. 1,1-Diphenyl-2-Picrylhydrazyl radical scavenging activity

Briefly, 2 mL of DPPH solution (0.1 mM) was mixed with 50 μL of extract. After shaking vigorously, the mixture was allowed to stand in the dark for 30 min, and absorbance was measured at 517 nm with a UV–Visible spectrophotometer (PG instruments, T80) against a blank of ethanol (38).

##### 2.2.1.2. Ferric ion reducing antioxidant power assay

Briefly, the freshly prepared FRAP reagent (2.08 mL) was mixed with 240 μL of distilled water and 80 μL of the test sample in the test tubes and vortexed. The absorbance of test samples was measured at 593 nm against a blank with a UV–Visible spectrophotometer (PG instruments, T80) (38).

#### 2.2.2. Total anthocyanin content

The pH differentiation method was used to determine the TAC of *P. avium* fruit (39). Briefly, the ethanolic fraction of sweet cherries was mixed with 0.4 M sodium acetate buffer (pH 4.5) or 0.025 M potassium chloride buffer (pH 1.0), and absorbance was measured within 20 to 50 min at 700 nm and 520 nm, respectively, with UV–Visible spectrophotometer (PG instruments, T80). Cyaniding-3-glucoside was used as a standard for estimating TAC, and results were expressed as mg C3G/g.

#### 2.2.3. Quantification of phytochemicals with HPLC

The ultrasonic extract of fruit was subjected to quantitative analysis to confirm the phenolics. Before subjecting the extract to HPLC analysis, the extract was hydrolyzed using the standard method (40). Gradient HPLC (LC-10A, Shimadzu, Japan) coupled with a C_18_ column and UV–visible detector was used to separate phenolic substances by keeping the flow rate of 1 mL/min. The identification of phenolic acids was performed by comparing retention time and spectra of the peak with previously injected standards, and quantification was established through external calibration.

### 2.3. *In vivo* study

A bio-efficacy trial was conducted to assess the anti-mutagenic potential of *P. avium* against DMBA-induced hepatocarcinogenesis. For this purpose, 40 male Wister albino rats weighing 180–200 g were procured. Acclimatization was provided for two weeks under optimal conditions, i.e., light (12 h light and dark cycle), temperature (27 ± 2°C), relative humidity (40–60%), and pathogen-free environment. During acclimatization, rats were supplied *ad libtium* water and feed intake. Rats (*n* = 40) were divided randomly into four groups, as mentioned in the treatment plan (Table 1). The hepatocarcinoma was induced through 7,12-dimethylbenz(a)anthracene procured from Tokyo Chemical Industries (TCI, Tokyo, Japan). Carcinogen was injected to experimental rats through the intraperitoneal route (1 mL syringe) mixed in corn oil (20 mg/Kg of B.W) once a week for eight weeks. The treatment group was supplemented with 500 mg/Kg of B.W of *P. avium* (sweet cherries) UAE intra-gastrically through gavage daily. Doxorubicin (2 mg/mL) procured from Pfizer Laboratories Ltd. Pakistan served as a chemotherapeutic agent. For this purpose, 0.54 mg/Kg dose of doxorubicin, equivalent to 20 mg/m^2^ of human dose, calculated according to Abd El-Moneim, Abd El-Rahim (41) was injected intravenously (lateral tail vein of rat) once a week throughout the experimental trial. At the end of the experimental trial, rats were decapitated by cervical dislocation to collect blood samples, and the liver was immediately preserved for further histopathological and gene analysis.

**Table 1:** Treatment plan for efficacy trial.

Groups	Treatment
HCG_0_: Normal control	Normal feed
HCG_1_: Negative control/model group	Normal feed + DMBA
HCG_2_: Standard group	Normal feed + DMBA+ Doxorubicin
HCG_3_: Treatment group	Normal feed + DMBA+ *Prunus avium* extract

#### 2.3.1. Physical parameters

The weight, feed and water intake of the experimental rats were recorded weekly throughout the trial. The weight of the liver was recorded at the time of decapitation.

#### 2.3.2. Biochemical parameters

Serum was analyzed for tumor biomarkers (AFP and CEA) using commercially available ELISA kits (42). Serum ALT and AST were determined using available Bioclin Diagnostic Kits (Germany) according to instructions provided by the manufacturer. However, serum LDH was assessed using commercial kits based on the conversion of lactate to pyruvate (43). Also, serum TOS and TAOC were assessed using the colorimetric method (44, 45).

#### 2.3.3. Ultrasonography

For Sonographic examinations of the experimental rats, an ECM EXAGO (France) system with a high-resolution linear rectal imago probe (5–10 MHz) and 7.5 MHz frequency was used. Rats were kept under anesthesia. Hair clippers depilated the abdominal region, alcohol and the usual acoustic coupling gel assisted the ultrasound transmission. Outlining of the boundaries of the plane was carried out for each nodule (46).

#### 2.3.4. Gross morphology and histopathology

Histology was done to illustrate the condition of hepatic tissues of DMBA-induced cancer-bearing rats. After decapitation, hepatic tissues were expurgated, and fixation with 10% formalin was done for 10 days. Afterwards, fixed tissues were dehydrated with the help of graded ethanol and after paraffin wax embedding, 5 μm thick sections of tissues were obtained by cutting with a rotary microtone. Staining of the tissue sections spread over glass slides was performed using hematoxylin and eosin dye. Finally, mounted and stained hepatic tissues were observed under the light microscope (BX51, Olympus Company, Japan) at 40X (47).

#### 2.3.5. Gene expression analysis

TRIzol reagent was used to extract total RNA (ThermoFisher Scientific, Massachusetts, United States) from hepatic tissues (48). After extraction, nanodropping was done for the quantification of RNA. The obtained RNA was then utilized for cDNA synthesis using a RevertAid cDNA synthesis kit (ThermoFisher Scientific) according to the manufacturer’s instructions.

Quantitative real-time PCR (qRT-PCR) was thus performed by using the Maxima SYBR Green/ROX Mater Mix kit (ThermoFisher Scientific). Gene analyzed for their expression has been provided in Table 2 along with their primers sequence. All the forward and reverse primers for required genes were provided by Synbio Technologies (NJ, United States). β-actin played the role of a housekeeping gene. During PCR quantification, the cDNA template was denatured for 15 s (95°C) for all the mentioned genes, followed by 30 s (58°C) annealing. Furthermore, the extension was recorded at 20 s for 39 cycles (72°C). The obtained data set was analyzed using the 2*(−ΔΔct) method (49).

**Table 2:** Source and name of primers (Oligonucleotides).

Source	Forward Sequence (5′ → 3)	Reverse Sequence (5′ → 3)
β-actin	CGAGTACAACCTTCTTGCAGC	TATCGTCATCCATGGCGAACTG
ATM	CACCTTAAGGGTTCTCGTCG	TCAGAACGTGCATCCTCACC
CHEK1	CACAGGAGGGAAGGCAACAT	TGAAGATAAACCACCCCCGC
CHEK2	GCATACGTGTGGGGTAGGA	CCCTGAAGATGCGAAAGTGC
CDKN1α	TGTGATATGTACCAGCCACAGG	CAGACGTAGTTGCCCTCCAG

### 2.4. Statistical analysis

Analysis of variance and *Post Hoc* Tukey test at a *p* ≤ 0.05 were performed for *in vivo* study parameters, i.e., physical parameters, biochemical parameters and gene expression analysis. Besides, origin pro and graph pad prism 8.0 were used for graphical representation.

## 3. Results

### 3.1. Antioxidant potential and phytochemical screening of *Prunus avium*

**Figure 1:**
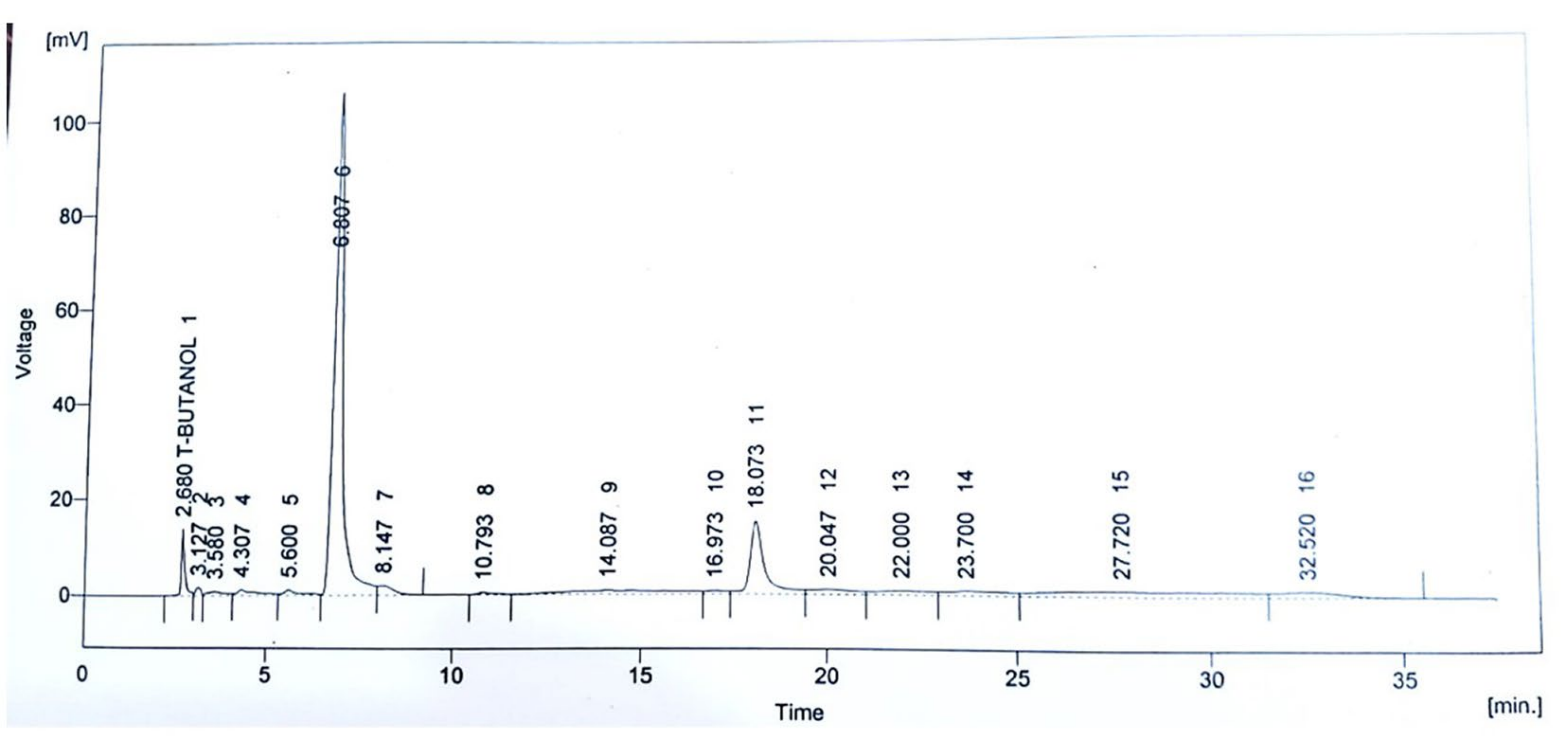
HPLC Chromatogram for quantification of polyphenols in ultrasound assisted extract of *P. avium.*

The phytochemical screening of *P. avium* extract was performed, which revealed an extract yield of 14.67 ± 0.35%. Moreover, the assessment of antioxidant potential of *P. avium* exhibited 74.10 ± 1.78% DPPH inhibition and 56.00 ± 1.34 μM Fe^2^+/g FRAP values. Besides, 1.98 ± 0.05 mg C3GE/g anthocyanin content was obtained. The HPLC quantified phenolics, ferulic acid, quercetin, gallic acid, syringic acid, p and m coumaric acid are given in Table 3, while, Figure 1 represents the HPLC chromatogram for said compounds.

**Table 3:** HPLC quantification of polyphenols in *P. avium* ultrasound assisted extract.

Phenolic compounds	Quantity (ppm)
Ferulic acid	5.8976
Quercetin	4.8862
p-Coumaric acid Gallic acid m-Coumaric acid	5.3247 1.5839 0.9598
Syringic acid	0.6431

### 3.2. Effect of *Prunus avium* extract on physical parameters

**Figure 2:**
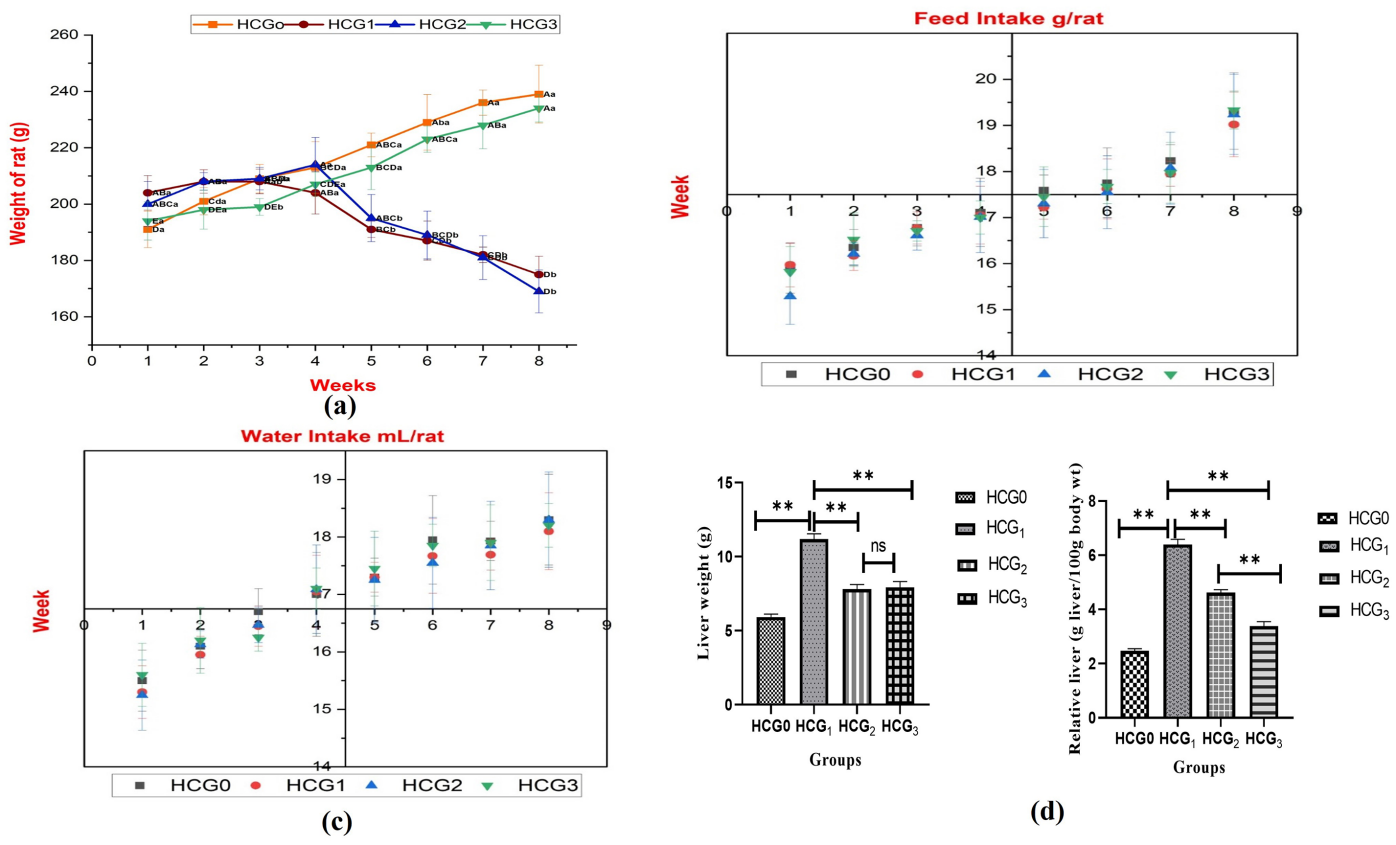
Physical parameters **(A)** Body weight (different letters designate significant variation between study groups and weeks. The capital letters designate differences among weeks in a group separately, whereas small letters represent differences among groups for weeks); **(B)** Feed intake; **(C)** Water intake: **(D)** Liver weight and relative liver weight of normal control (HCGo), negative control (HCG_1_ = DMBA), standard group (HCG_2_ = DMBA+Doxorubicin) and treatment group (HCG_3_ = DMBA+P. avium extract). NS = Non-significant (*p* > 0.05); * = Significant (*p* < 0.05); ** = Highly significant (*p* < 0.01).

The weight of rats was significantly different (*p* < 0.01) among all the groups. The highest decline in body weight was observed in doxorubicin-treated rats, followed by diseased rats. However, the administration of UAE significantly maintained a healthy weight, as depicted in Figure 2A. Treatments exerted a nonsignificant effect on feed and water intake. The feed intake varied between 15.29 ± 0.61–19.33 ± 0.41 g/rat, and water intake ranged between 15.25 ± 0.46–18.30 ± 0.79 mL/rat (Figures 2B,C). The hepatic weight was assessed at the time of decapitation as alterations in it can help to detect and evaluate the effects of hepatotoxins. Hepatic weight was significantly (*p* < 0.01) different among all the groups, but doxorubicin-treated rats exhibited the highest maintenance of healthy hepatic weight, followed by extract-fed rats. The relative liver weight followed a similar trend, but extract-fed rats demonstrated the lowest relative liver weight, followed by doxorubicin-treated rats. Hepatic weight and relative liver weight were higher in untreated diseased rats.

### 3.3. Effect of *Prunus avium* extract on tumor biomarkers

**Figure 3:**
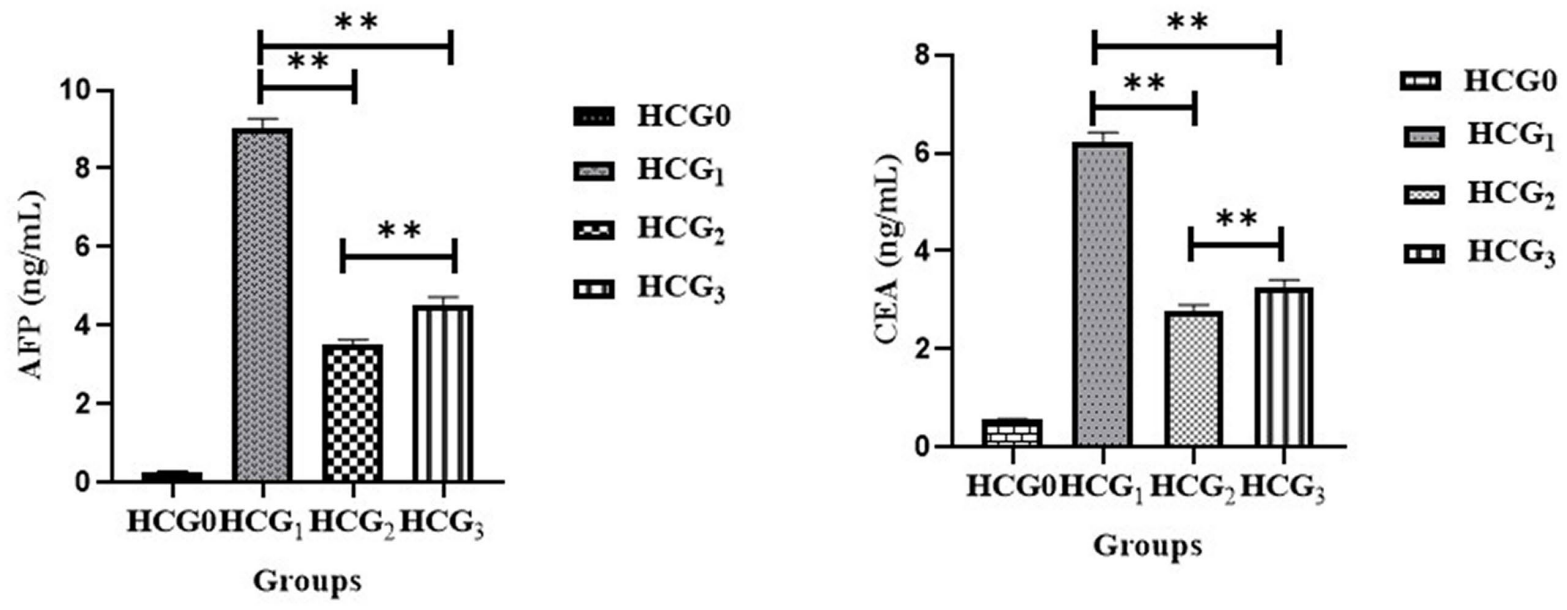
Serum AFP and CEA (ng/mL) (±SD) of normal control (HCGo), negative control (HCG_1_ = DMBA), standard group (HCG_2_ = DMBA+Doxorubicin) and treatment group (HCG_3_ = DMBA+*P. avium* extract). NS = Non-significant (*p* > 0.05); * = Significant (*p* < 0.05); ** = Highly significant (*p* < 0.01).

The antiproliferative potential of *P. avium* polyphenols was assessed by measuring the concentrations of AFP and CEA in rat sera. The extract administration significantly (*p* < 0.01) attenuated the raised AFP and CEA levels (Figure 3) as compared to the diseased rats. The highest decline in levels of tumor biomarkers was observed in the doxorubicin-treated rats followed by UAE-fed rats.

### 3.4. Effect of *Prunus avium* extract on hepatic enzymes

**Figure 4:**
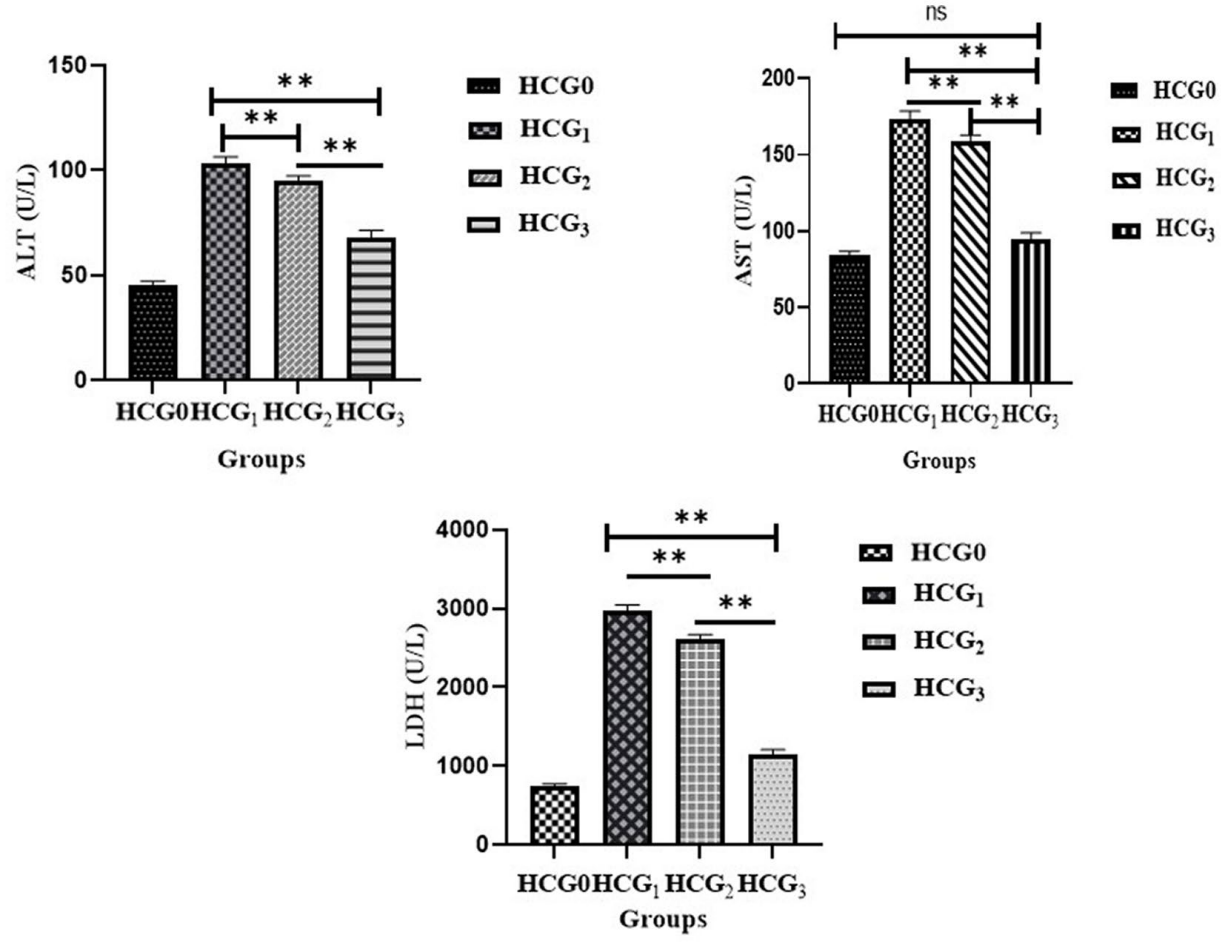
Serum ALT, AST, and LDH (U/L) (±SD) of normal control (HCGo), negative control (HCG_1_ = DMBA), standard group (HCG_2_ = DMBA+Doxorubicin) and treatment group (HCG_3_ = DMBA+*P. avium* extract). NS = Non-significant (*p* > 0.05); * = Significant (*p* < 0.05); ** = Highly significant (*p* < 0.01).

The hepatic transaminase activities were assessed in rats’ sera to evaluate the hepatoprotective impact of *P. avium* polyphenols. A significant (*p* < 0.01) decline in levels of ALT, AST and LDH (Figure 4) was elucidated in HCG_3_ rats. However, the model group exhibited a significant rise in serum ALT, AST and LDH levels.

### 3.5. Effect of *Prunus avium* extract on oxidative stress biomarkers

**Figure 5:**
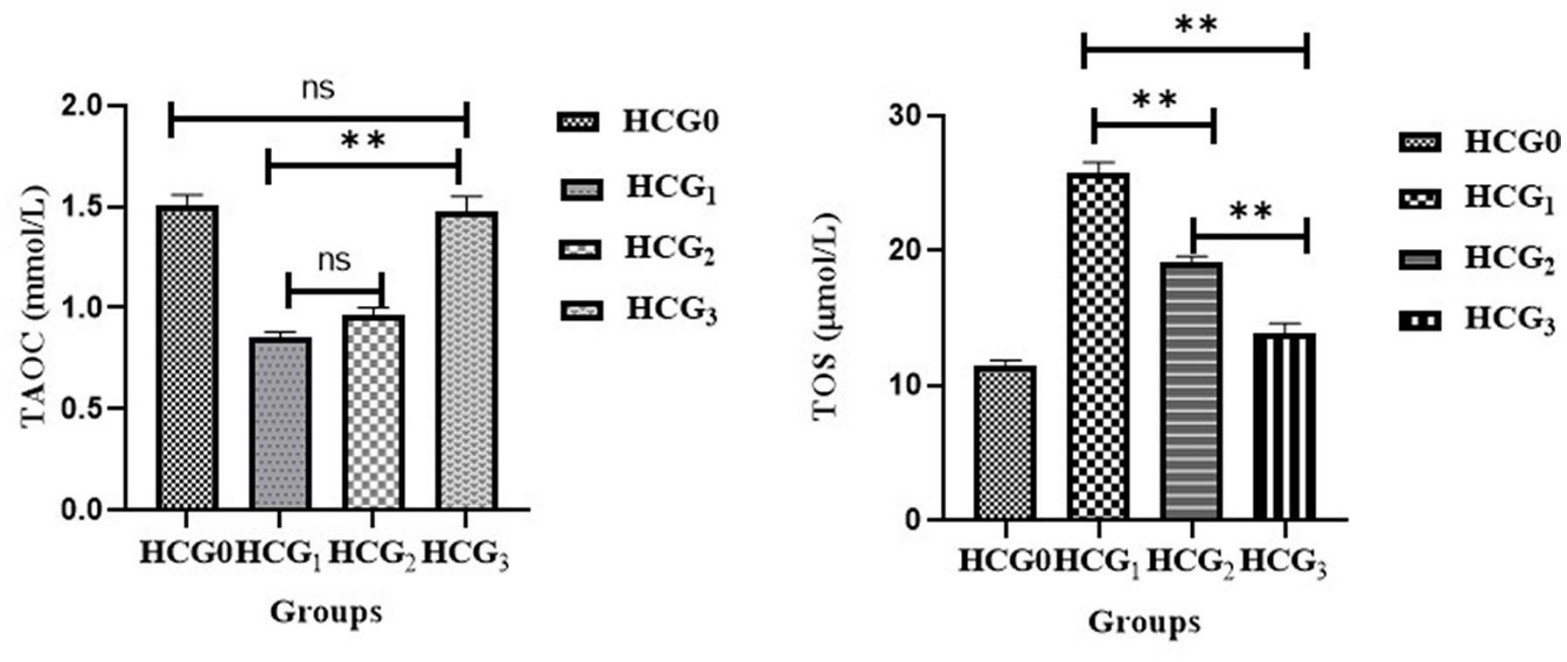
Serum total antioxidant activity (mmol/L) and total oxidative stress (μmol/L) levels (±SD) of normal control (HCGo), negative control (HCG_1_ = DMBA), standard group (HCG_2_ = DMBA+Doxorubicin) and treatment group (HCG_3_ = DMBA+*P. avium* extract). NS = Non-significant (*p* > 0.05); * = Significant (*p* < 0.05); ** = Highly significant (*p* < 0.01).

The levels of TAOC and TOS in rats’ sera were assessed to observe the impact of *P. avium* extract on oxidative stress. Diseased rats showed a significant (*p* < 0.01) upsurge in TOS levels. The highest decline in serum TOS was observed in UAE-fed rats. Similarly, extract-fed rats exhibited the marked rise (*p* < 0.01) in serum TAOC due to phytochemicals, followed by the doxorubicin-treated group. However, a significant drop in the TAOC was monitored in cancer-bearing untreated rats (see Figure 5).

### 3.6. Effect of *Prunus avium* extract on hepatic ultrasonography

**Figure 6:**
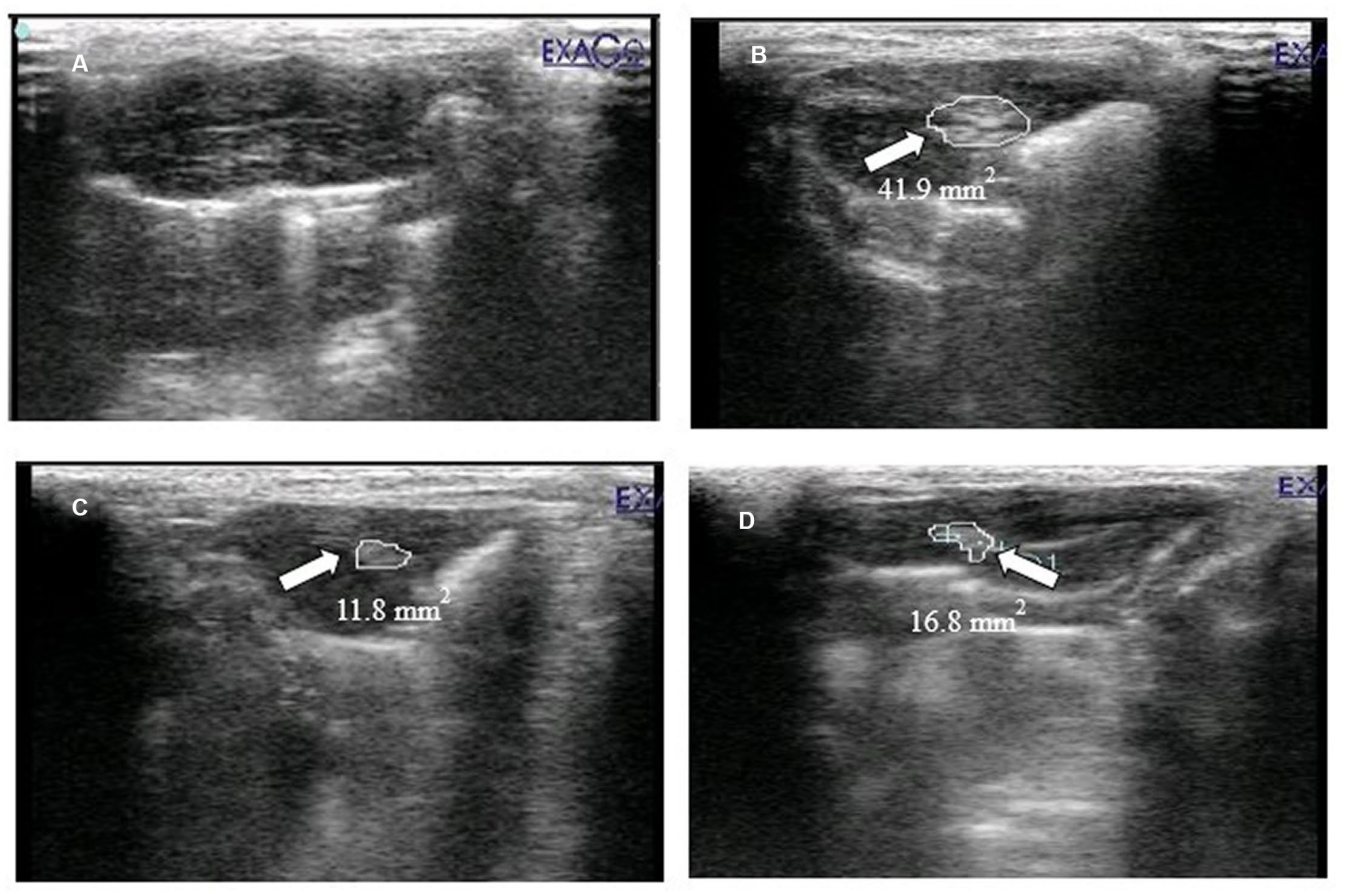
Ultrasound imaging of **(A)** normal control (HCGo), **(B)** negative control (HCG_1_ = DMBA), **(C)** standard group (HCG_2_ = DMBA+Doxorubicin) and **(D)** treatment group (HCG_3_ = DMBA+*P. avium* extract).

The HCC was confirmed by scanning the liver of experimental rats using ultrasonography. The sonographic image of normal control is given in Figure 6A. The hepatic ultrasound of diseased rats (Figure 6B) showed a round and well-defined target lesion nodule. It also exhibited irregular heterogeneous hyperechoic mass surrounded by a hypoechoic halo. While Figure 6C (standard group rats) and Figure 6D (extract-fed rats) demonstrate the attenuating effect of both treatments.

### 3.7. Effect of *Prunus avium* extract on hepatic histopathology

**Figure 7:**
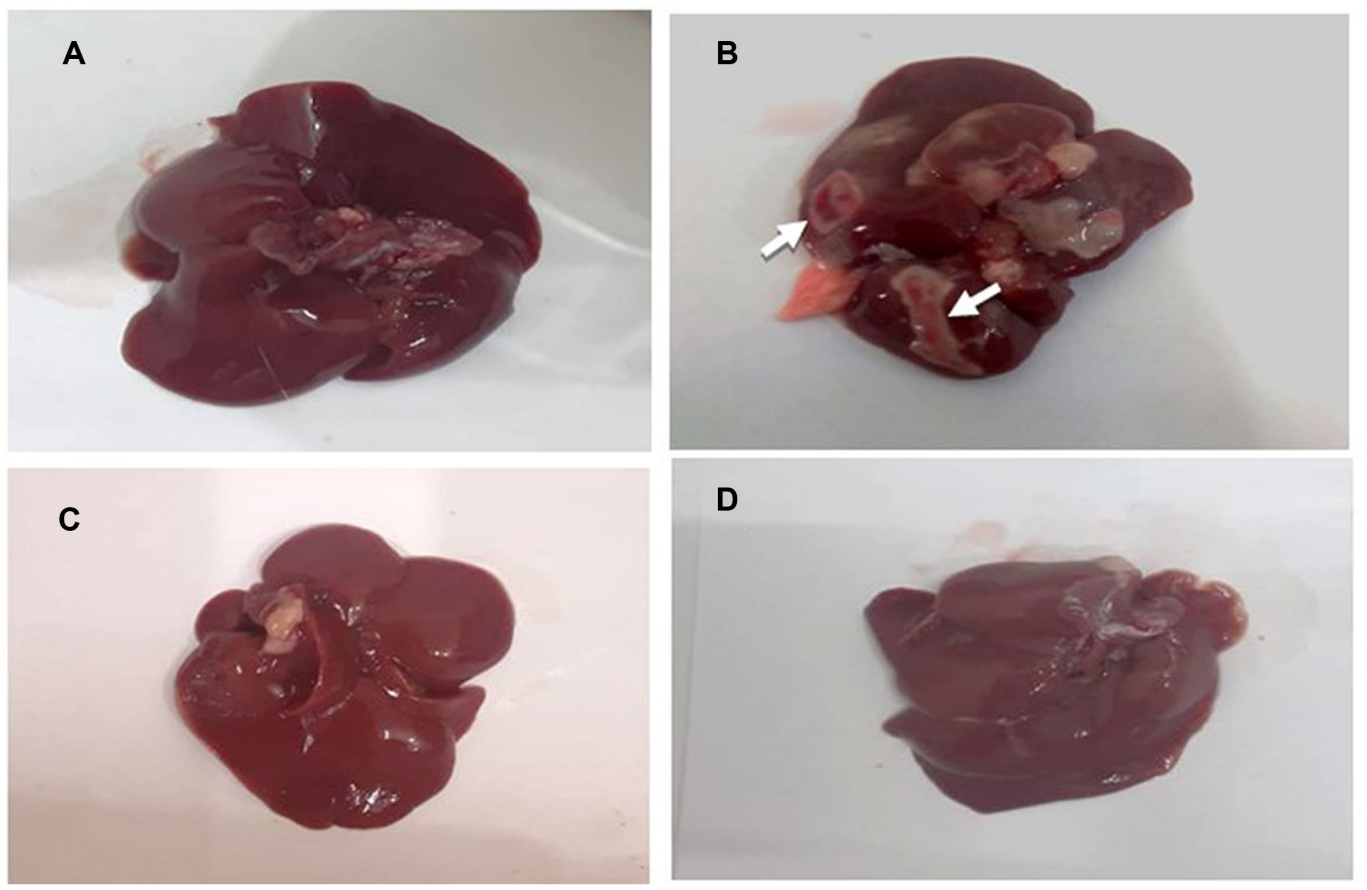
**(A–D)**: Gross anatomy of rat’s liver. **(A)** normal control **(B)** negative control (HCG_1_ = DMBA), **(C)** standard group (HCG_2_ = DMBA+Doxorubicin) and **(D)** treatment group (HCG_3_ = DMBA+*P. avium* extract).

The gross morphology of the liver of experimental rats is presented in Figures 7A–D. The liver of the normal control and standard group rats (HCG_2_) were bright dark reddish brown in color with smooth surface (Figures 7A,C). Figure 7B demonstrates the gross anatomical changes in diseased rats (HCG_1_) in which liver had the presence of nodule like structures along with coarse surface and hepatic discoloration as compared to normal control. However, slight discoloration was observed in liver of *P. avium* UAE fed rats (HCG_3_) though hepatic surface was smooth (Figure 7D).

**Figure 8:**
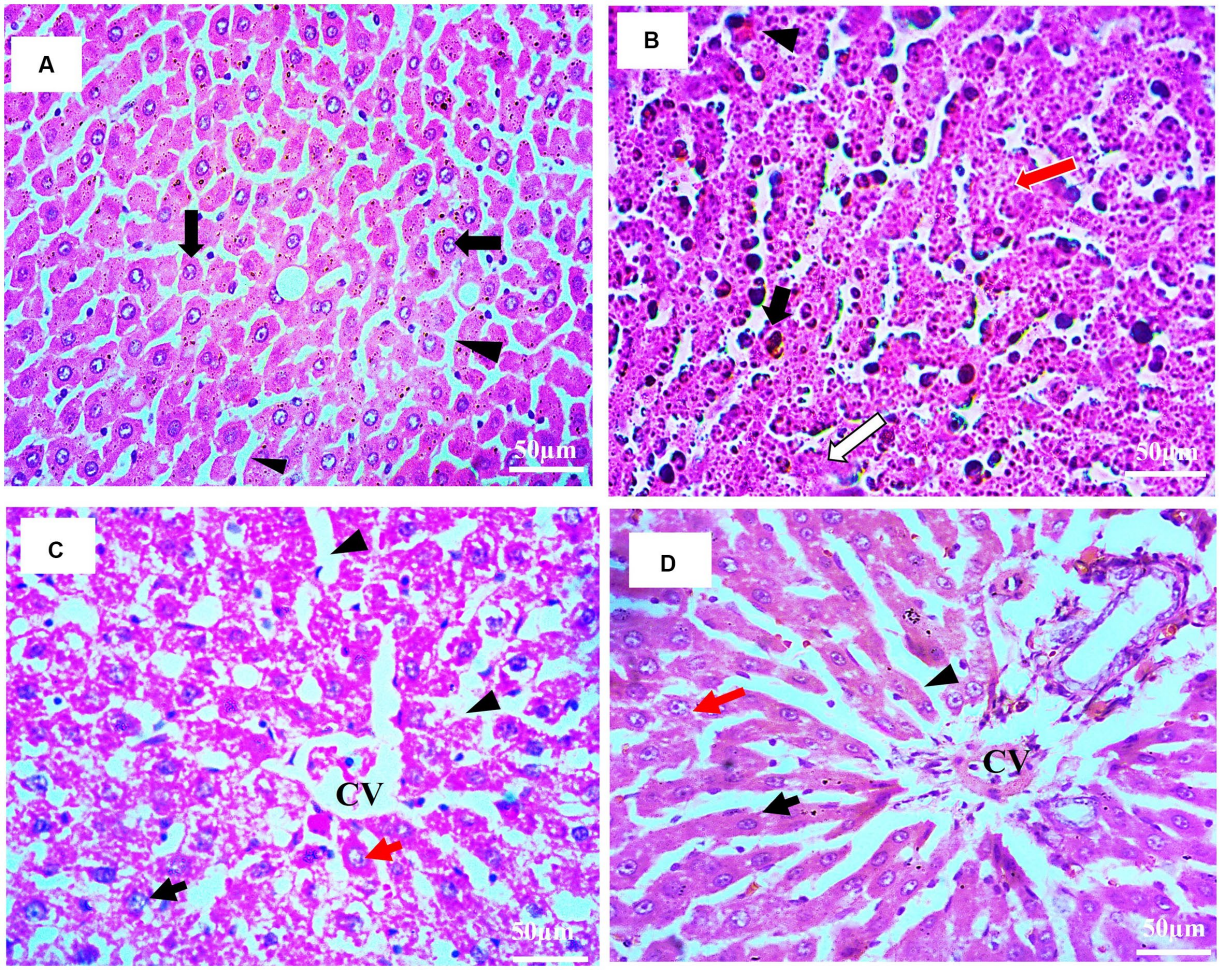
**(A–D)**: Histopathological examination of rat’s liver (H&E staining; 40X). **(A)** Histopathology of normal rat’s liver tissue (HCGo): hepatocytes with centered nuclei (black arrow) and sinusoidal spaces (arrowhead) **(B)** histopathology of HCC bearing rat’s liver tissue (HCG_1_): nests of hepatocytes (red arrow), necrosis (white arrow), hemosiderin macrophages (black arrows); congestion (black arrow head) **(C)** histopathology of drug treated rat’s liver tissue (HCG_2_): central vein (CV); nucleus (black arrow); sinusoidal spaces (arrowhead); hepatocytes (red arrow) **(D)** histopathology of *P. avium* fruit extract treated rat’s liver tissue (HCG_3_): central vein (CV); nucleus (black arrow); sinusoidal spaces (arrowhead); hepatocytes (red arrow).

In compliance with gross morphology, the histopathological examination of the hepatic tissues of experimental groups is represented in Figures 8A–D. The histopathology of liver tissues of normal control (Figure 8A) demonstrated hexagonal hepatocytes with central nuclei. Hepatic parenchyma and sinusoidal spaces were intact. The histopathology of the negative control (HCG_1_) rat’s liver tissues (Figure 8B) clearly showed pleomorphism, loss of lobular structure, and abnormal trabeculae to sinusoidal arrangements. Nests of hepatocytes with mixed growth patterns and hemosiderin macrophages were also present. Furthermore, necrotic debris accumulation was also observed. The photomicrograph of liver tissue for standard group is displayed in Figure 8C where hepatocytes with prominent nuclei were radiating from the central vein. Mild dilations in blood sinosoides with minimal congestion were also seen. The histopathological examination of treatment group rats (Figure 8D) indicated moderate damage in hepatic parenchyma cells along with moderate sinusoidal dilation. Furthermore, necrotic changes were reduced as compared to model group. Thus, extract supplementation prevented the neoplastic nests’ formation followed by the repairing of the tissues.

### 3.8. Effect of *Prunus avium* extract on ATM cascade

**Figure 9:**
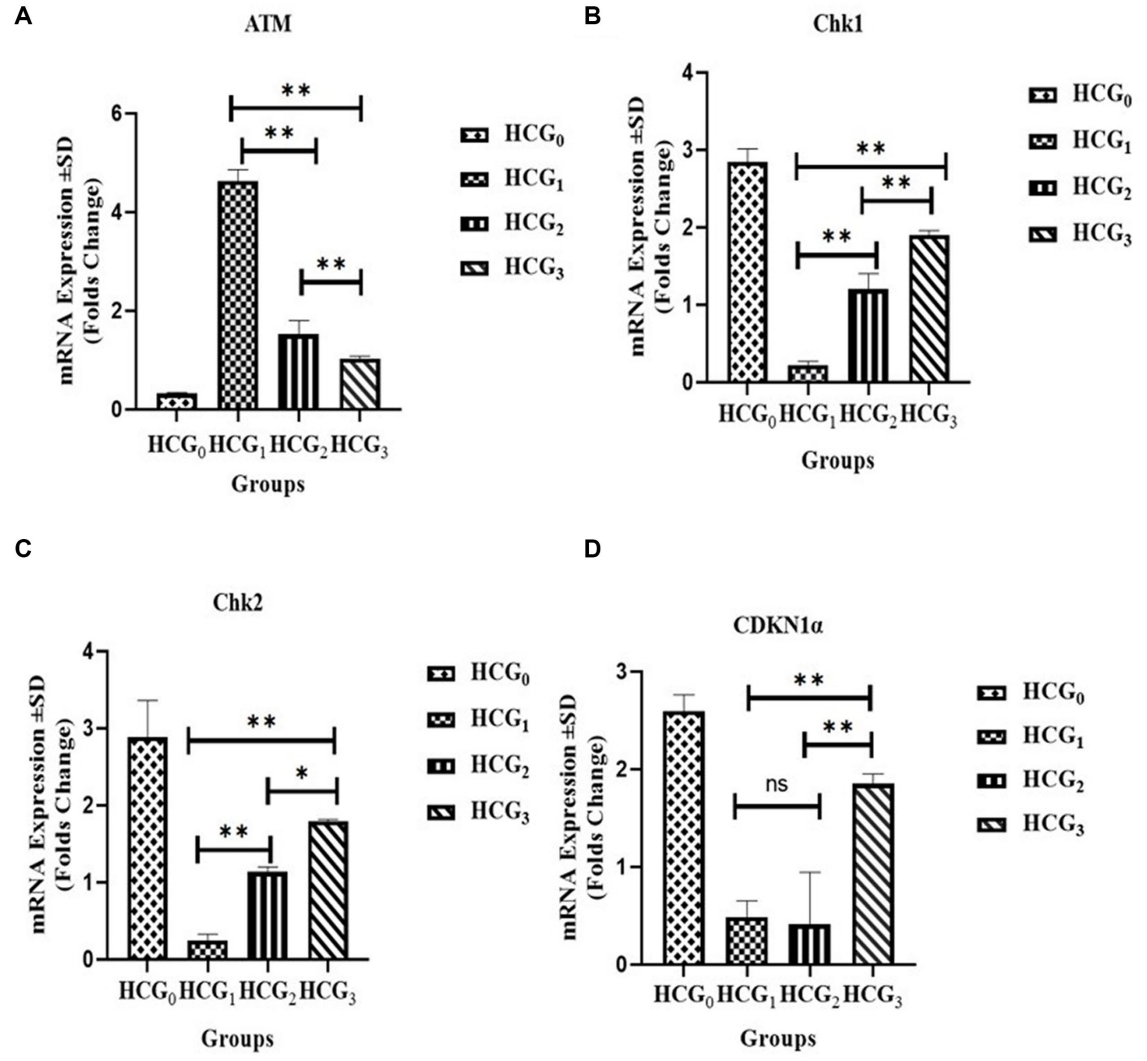
Gene expression analysis. mRNA expression of **(A)** ATM: **(B)** CHEK1: **(C)** CHEK2 and **(D)** CDKN1α (±SD) of normal control (HCGo), negative control (HCG_1_ = DMBA), standard group (HCG_2_ = DMBA+Doxorubicin) and treatment group (HCG_3_ = DMBA+P. avium extract). NS = Non-significant (*p* > 0.05); * = Significant (*p* < 0.05); ** = Highly significant (*p* < 0.01).

The uncontrolled cell cycle is a hallmark of tumors contributing to cancer progression and invasion. Furthermore, the cell checkpoints, specifically G_2_, halt the cell’s progression into the mitosis phase, thereby allowing cells to repair and stop the proliferation of cancerous cells when DNA damage exists. The mRNA expressions of the ATM gene, CHEK2, CDKN1α(p21^CIP/WAF1^) gene, and CHEK1 gene were monitored for the ATM signaling pathway. The mRNA expression levels of CHEK1, CHEK2, and CDKN1α(p21^CIP/WAF1^) genes were significantly (*p* < 0.01) higher in the extract-fed rats as compared to cancer-bearing untreated rats (Figure 9) exhibiting reduced expression of aforementioned genes. However, mRNA expression levels of ATM gene were downexpressed in the extract-fed and standard groups, than diseased rats which showed significantly higher mRNA expression of said gene.

## 4. Discussion

Owing to the significant rise in cancer incidence, developing novel treatments is becoming essential. Antineoplastic therapies have frequently involved administering anti-apoptotic drugs known for inhibition of proliferation, invasion, and metastasis. Dietary interventions are the main approach For preventing cancer development and enhancing survival rates (50, 51). Also, synthetic antineoplastic therapies are barely free of detrimental effects and impose a financial burden that has increased the researcher’s interest in alternative options (52). Evidences show that a diet rich in natural bioactive metabolites could reduce liver cancer risk. Polyphenols strictly perform the role of antioxidants through the induction of apoptosis and halting the events involved in proliferation, angiogenesis, invasion and metastasis. Carcinogenesis induced by chemicals is forcibly associated with excessive generation of ROS (reactive oxygen species), eliciting time-dependent necrosis by increasing the number of oxidized thiol proteins (28, 53).

Phytochemical screening of *P. avium* fruit extract revealed anthocyanins, ferulic acid, quercetin, m coumaric acid, syringic acid, gallic acid, and p coumaric acid as major bioactive metabolites. Flavonoids as well as phenolics are deemed valuable because of their indorsed antioxidant potential. Furthermore, their free radical scavenging potential augments their significance under oxidative stress, inflammatory disorders and cancer. Owing to cancer progression and its subsequent treatment, the body can display dramatic alterations in weight and composition. Cancer-based weight loss accompanies an equal loss of adipose tissues as well as skeletal muscle mass and affects 30–80% of cancer patients. Numerous cancers are correlated with cachexia, which embodies weight loss improperly impacting the lean body mass. It is usually attributed to the inflammatory cytokines associated with disease burdens, such as TNFα, IL-6, and IL-1α. It is considered an essential prognostic factor in neoplasm as the higher the rate of weight loss shorter is the survival time (54, 55). In the current trial, the UAE of *P. avium* prevented weight loss in rats as compared to the drug-treated and the diseased rats.

Furthermore, the maintenance of hepatic weight in the healthy range was also achieved through extract feeding. Since a significant gain in hepatic weight can be caused by the accumulation of lipids, glycogen or other substances or by cellular damage, congestion, hepatocellular hypertrophy, or hyperplasia, primary or secondary neoplasia can also be a reason for alterations in it. However, loss in hepatic weight is unusual and might be attributed to loss of functional mass associated with atrophy or lethal hepatocellular injury (acute injury) (56). The AFP and CEA are considered significant tumor biomarkers that have been reported to be increased in HCC-bearing rats. Also, AFP can regulate tumor growth, and suppressing it may be an effective treatment (57). In the current study, the oral administration of sweet cherries UAE dropped the concentrations of these tumor biomarkers.

DMBA could induce hepatic injury, resulting in the deterioration of cell membranes and ultimately causing the release of transaminases from hepatic tissues. Hepatic enzymes, including ALT, AST and LDH are major indicators of hepatic injury. LDH has been considered a metastasis marker, particularly in the liver and an independent, crucial prognostic indicator, i.e., the higher the LDH lesser the survival rate. Furthermore, the elevated LDH and raised AST and ALT concentrations are associated with massive hepatocyte damage. Also, elevated levels of LDH associated with elevated alkaline phosphatase (AP) suggest malignant hepatic infiltration (58). In the current trial, the UAE administration reduced AST, ALT and LDH as compared to the model group. The findings indicate that extract administration reduces liver transaminases by helping hepatic regeneration and maintaining the integrity of the cellular membrane. Thus, sweet cherries UAE possesses anti-mutagenic potential against DMBA-induced HCC. These findings are further supported by Cheng, Wang (59) and Li, Li (60) where mulberries polysaccharides and whole powder prevented the *in vivo* hepatocarcinogenesis. Also, sweet cherries exhibited reduced LDH oxidation and nitric oxide generation in Caco-2 cancer cells (61). Treatment of HepG_2_ cells with sweet cherry extract attenuated the LDH leakage (35). Besides, Song, Wu (36) further elaborated the protective effect of sweet cherries in hepatic steatosis-bearing rats. Phytochemicals alleviate necrotic cell death and protect against hepatic damage through the restoration of the cellular antioxidant defense system, modulation of oxidative stress, and consequently mitigate mitochondrial dysfunction and inflammation. Furthermore, they upregulate hepatic cholesterol 7α-hydroxylase expression and activate the potential LXRα-CYP7A1-bile acid excretion pathway (62, 63). This disease attenuating potential of sweet cherries may be attributed to these protective roles of phytochemicals.

The normal reactive oxygen species production (ROS), generation of hydrogen peroxides (H_2_O_2_), hydroxyl radicals as well as reactive nitrogen species is essential for tissue homeostasis and normal cellular functioning (13, 14). But any abnormality in their production or accumulation leads to oxidative stress and subsequent cellular damage. The continuous upshot of ROS has been linked to tumor onset, growth, proliferation as well as aggressiveness (64). DMBA induction resulted in raised TOS levels that were significantly dropped in extract-fed rats. A previous study by Wang, Li (65) assessing the antioxidant and anti-apoptotic effect of *Prunus yedoensis* (cherry blossom) fraction concluded a noticeable decline in reactive oxygen species and malondialdehyde levels. Also, bilberry extract significantly lowered the ROS production in hydrogen peroxide-treated AML12 cells by providing resistance against oxidative stress (66). The malvidin-3-galactoside from blueberries demonstrated the marked reduction in oxidative stress through suppressing the accumulation of ROS in Huh-7 HCC cell lines (67). Also, sweet cherry phenolic-rich fractions and isolated phenolics protected the MDCK-MDR1 and MDCK-II cells from the H_2_O_2_ and tert-butyl hydroperoxide-induced oxidative stress (68). Apart from reducing oxidative stress, extract administration significantly enhanced the TAOC in the current trial. Antioxidants can delay the oxidative process by acting as key inhibitors of initiation as well as propagation of oxidizing chain reactions (69).

Furthermore, histopathological examinations confirmed the pleomorphism, necrosis and formation of neoplastic lesions in DMBA-induced HCC-bearing rats. However, the *P. avium* fruits extract exerted a regenerative and preventive role as cherries possess higher concentrations of anthocyanins than other bioactive metabolites, which enhance their biological potential mainly due to multiple hydroxyl groups on the B ring (24, 31). Apart from anthocyanins, several non-colored phenolics such as hydroxycinnamic acids and quercetin derivatives have also been reported to exert anticancer potential against several human cancer cells (70–72). It is well known that phenolics have the structural capabilities for donating their hydrogen to radical species, thereby diminishing their effectiveness (12). Furthermore, the interaction among different phenolic sub-classes also enhances biological potential. Also, these moieties can cross the membrane barriers, thus scavenging the free radicals before their activity can initiate cellular damage and promote apoptosis (73, 74). Multiple hydroxyl group-based substitutions in the structure of phenolics can cause a pro-oxidant effect, thus intensifying the cytotoxic efficiency and inhibiting tumor cell growth (35, 75).

DNA injury is considered another critical mechanism involved in HCC initiation and progression. The arrest of the cell cycle at the G_2_/M phase indicates that intracellular DNA damage is difficult to repair. DMBA can result in DNA damage by generating double strand breaks (DSBs) in a dose dependent manner in extra-ovarian tissues (liver, peripheral lymphocytes, and skin cells) (76, 77). The DNA damage repair system (DDR) activates to repair as well as modulate the DNA damage, where the cellular checkpoints are the key regulatory mechanism. The cell checkpoints, specifically G_2_, halt the cell’s progression into the mitosis phase, thereby enabling cells to repair and stop the proliferation of cancerous cells when DNA damage exists (78–80). ATM/ATR cascade is widely believed to be activated because of intracellular DNA damage. Phosphorylation of DDR sensors (ATM and ATR), owing to DNA damage, inhibits the cell cycle through the activation of cellular checkpoints. The phosphorylation of Chk1 and Chk2, in turn, phosphorylate Cdc25C and inhibit their expression, thus, delaying the progression of the cell cycle at G_2_/M phase through the prevention of dephosphorylation of residues of CDK1 by keeping CDK1- Cyclin B1 complex in the inactivated state (78, 81, 82). Apart from this, cyclin kinases inhibitors (p21 and p27) also control the transition of G_2_/M phase by halting the activity of cyclin B1/Cdc2 complex (83, 84). Also, p21 activation is associated with cell cycle arresting at G_1_/S phase. This cell cycle arresting at G_1_/S and G_2_/M phase is crucial to prevent the passing of unpaired DNA mutations to daughter cells (85). Decreased expression of Cdc2 and increased expression of its form (non-active (Tyr15)-phosphorylated Cdc2) frequently exist at G2/M phase arrest and senescence (86). Also, autophosphorylated ATM leads to the activation of BRCA1 and ATF2, thereby promoting the DNA repair signaling cascades involving hundreds of sensors, transducers, and effectors (85). Several *in vivo* studies reported the role of plant-based phytoceuticals in cancer prevention *via* cell cycle arrest. Goji berries promoted the blocking of cancerous cells at G_1_ phase and induced apoptosis (87). Shen et al. (10) reported the arresting of cell cycle at M/G_2_ phase of HepG_2_ cells *via* inhibition of expression of Cdc25c by treating cancerous cells with *Prunus persica*. The anthocyanin rich blueberries extract arrested the cell cycle at G_2_/M phase and induced apoptosis in oral cancer cell lines in dose dependent manner (88). Conclusively, *P. avium* UAE upregulated the downstream targets of ATM signaling. Based on the findings of serum analysis, histopathology and gene expression analysis, *P. avium* fruit extract possesses the anti-oxidative, anti-inflammatroy and anticancer potential against chemical induced physiological alterations in hepatocarcinoma.

## 5. Conclusion

The current research provides scientifical evidence of the hepato-protective, anti-oxidative, anti-inflammatory and anti-cancer potential of sweet cherries polyphenol-rich fraction. Our findings demonstrated that *P. avium* extract exerted anticancer and anti-mutagenic potential *via* reduction of raised tumor markers as well as hepatic transaminases and enhancement of antioxidant capacity followed by increased expression of cellular checkpoint kinases (1 and 2) and P21, thereby reducing oxidative stress, hepatic malfunctioning, tumor cell growth and proliferation. Bio-equivalency among rodents and humans is unknown owing to species as well as metabolic rate differences. So, further studies in human subjects with HCC are required to ascertain the degree and spectrum of sweet cherry-derived clinical benefits.

## Data availability statement

The original contributions presented in the study are included in the article/Supplementary material. Further inquiries can be directed to the corresponding author.

## Ethics statement

The animal handling protocols used in current research work were approved by guidelines of National Biosafety Rules 2005, Punjab Biosafety Rules 2014, and Bioethics Protocols, Office of Research, Innovation, and Commercialization (ORIC), University of Agriculture, Faisalabad Pakistan.

## Author contributions

RS: investigation, methodology, formal analysis, data curation, conceptualization, and writing–original draft. MK: conceptualization, funding acquisition, validation, supervision, visualization, and project administration. MB: conceptualization, writing–review and editing, supervision, and resources. MF: conceptualization, writing–review and editing, supervision, resources, and software. All authors contributed to the article and approved the submitted version.

## Funding

The work has been sponsored by the Higher Education Commission under the project “Indigenous 5000 Fellowship Program”.

## Conflict of interest

The authors declare that the research was conducted in the absence of any commercial or financial relationships that could be construed as a potential conflict of interest.

## Publisher’s note

All claims expressed in this article are solely those of the authors and do not necessarily represent those of their affiliated organizations, or those of the publisher, the editors and the reviewers. Any product that may be evaluated in this article, or claim that may be made by its manufacturer, is not guaranteed or endorsed by the publisher.
